# Human Engineered Cardiac Tissues Created Using Induced Pluripotent Stem Cells Reveal Functional Characteristics of BRAF-Mediated Hypertrophic Cardiomyopathy

**DOI:** 10.1371/journal.pone.0146697

**Published:** 2016-01-19

**Authors:** Timothy J. Cashman, Rebecca Josowitz, Bryce V. Johnson, Bruce D. Gelb, Kevin D. Costa

**Affiliations:** 1 The Cardiovascular Research Center, Icahn School of Medicine at Mount Sinai, New York City, New York, United States of America; 2 The Mindich Child Health and Development Institute, Icahn School of Medicine at Mount Sinai, New York City, New York, United States of America; Scuola Superiore Sant'Anna, ITALY

## Abstract

Hypertrophic cardiomyopathy (HCM) is a leading cause of sudden cardiac death that often goes undetected in the general population. HCM is also prevalent in patients with cardio-facio-cutaneous syndrome (CFCS), which is a genetic disorder characterized by aberrant signaling in the RAS/MAPK signaling cascade. Understanding the mechanisms of HCM development in such RASopathies may lead to novel therapeutic strategies, but relevant experimental models of the human condition are lacking. Therefore, the objective of this study was to develop the first 3D human engineered cardiac tissue (hECT) model of HCM. The hECTs were created using human cardiomyocytes obtained by directed differentiation of induced pluripotent stem cells derived from a patient with CFCS due to an activating BRAF mutation. The mutant myocytes were directly conjugated at a 3:1 ratio with a stromal cell population to create a tissue of defined composition. Compared to healthy patient control hECTs, BRAF-hECTs displayed a hypertrophic phenotype by culture day 6, with significantly increased tissue size, twitch force, and atrial natriuretic peptide (ANP) gene expression. Twitch characteristics reflected increased contraction and relaxation rates and shorter twitch duration in BRAF-hECTs, which also had a significantly higher maximum capture rate and lower excitation threshold during electrical pacing, consistent with a more arrhythmogenic substrate. By culture day 11, twitch force was no longer different between BRAF and wild-type hECTs, revealing a temporal aspect of disease modeling with tissue engineering. Principal component analysis identified diastolic force as a key factor that changed from day 6 to day 11, supported by a higher passive stiffness in day 11 BRAF-hECTs. In summary, human engineered cardiac tissues created from BRAF mutant cells recapitulated, for the first time, key aspects of the HCM phenotype, offering a new *in vitro* model for studying intrinsic mechanisms and screening new therapeutic approaches for this lethal form of heart disease.

## Introduction

Hypertrophic cardiomyopathy (HCM) is the abnormal thickening of the myocardium due to cardiomyocyte enlargement. It is a pathological cardiac disorder with no distinct precipitating insult, usually associated with a variety of genes encoding sarcomeric components [1]. Molecularly, HCM is characterized by enhanced expression of hypertrophic markers and aberrant calcium handling [2,3]. HCM remains one of the leading causes of death in young athletes [4] and it is rare for patients with HCM to remain hemodynamically stable. Most patients will develop some degree of obstructive disease that can lead to heart failure, while others experience arrhythmias and sudden cardiac death [5].

Animal models for HCM have been invaluable in developing an understanding of the mechanisms governing the development of the disease [6,7]. However, a key barrier to clinical translatability is the lack of species specificity. Two-dimensional human cell culture models of HCM are species-specific [8] but lack biofidelity and do not measure contractility. Therefore, a model system that recapitulates key aspects of human cardiac physiology, while maintaining species specificity, would benefit the development of novel therapeutics for HCM. Human engineered cardiac tissues (hECTs) offer a potentially useful strategy for understanding mechanisms of HCM since they reduce the biological complexity of the system to a manageable number of controllable factors while maintaining high biofidelity with direct measurement of electromechanical muscle function [9]. However, while enhanced mechanical loading has been shown to induce pathological hypertrophy in engineered tissues constructed from rat cells [10], and engineered tissues have been constructed using cells from murine models of HCM [11], no hECT models of human HCM currently exist. One explanation for this lack of models is the difficulty of creating human tissues with the acquired disease.

Alternatively, understanding inherited forms of HCM may lead to a greater understanding of the acquired form [12]. The RASopathies are a spectrum of disorders with aberrant activation of the RAS/mitogen-activated protein kinase (MAPK) pathway generally due to heterozygous germ-line mutations affecting these pathways; they constitute one of the most common groups of malformation syndromes [13]. The RAS/MAPK pathway is essential in regulating cell cycle, cell growth, differentiation and senescence. Congenital cardiac abnormalities, including HCM, are present in many of the RASopathies and may be due to the effect of abnormal RAS signaling in multiple populations of cardiac cell progenitors [12]. Furthermore, abnormal activation of the MAPKs has been found to lead to the hypertrophic response in animal models [14–17]. Recently, cardiac myocytes were derived from the induced pluripotent stem cells (iPSCs) of a patient with Noonan syndrome with multiple lentigines (formerly, LEOPARD syndrome) and found to be intrinsically hypertrophic [8]. Prior to this finding, it was not clear if the HCM in RASopathy patients was due to increased hemodynamic demands or intrinsic to the alterations in cardiomyocyte development. Thus, by studying the RASopathies, it is possible to develop a deeper understanding of the molecular pathways governing HCM development.

Cardio-facio-cutaneous syndrome (CFCS) is one of the RASopathies and is characterized most commonly by genetic mutations in *MEK1*, *MEK2* or *BRAF*. Cardiac abnormalities are among the most common findings in CFCS [18], including HCM in approximately 40% of patients [19]. Nearly 75% of patients with CFCS exhibit mutations in *BRAF*, which encodes a serine/threonine kinase and a direct effector of Ras [13]. CFCS patients with *BRAF* mutations have a higher prevalence of HCM versus the less common *MEK* mutations [20]. These data suggest BRAF may be an important component of the molecular pathway driving HCM.

The objective of this study was to create functional hECTs using iPSC-derived cardiac myocytes (iPSC-hECTs) from BRAF-mutant cells collected from a patient with CFCS and evidence of HCM and to examine functional and physical characteristics compared to wild-type (WT-) hECTs derived from healthy patients in order to assess how BRAF-hECTs recapitulate key features of HCM. We report that the BRAF-mutant hECTs exhibited several structural, molecular, and functional indications of a hypertrophic phenotype within one week of culture. While some distinctions from the WT-hECTs diminished over time, significant differences in twitch dynamics were maintained, potentially reflecting aberrant calcium handling in BRAF-hECTs. Principal component analysis and *k-*means clustering revealed diastolic force as a key factor that changed with time in culture. Overall, the study demonstrates the feasibility of combining tissue engineering and iPSC technologies to create a 3-D *in vitro* model of diseased human cardiac muscle mimicking key features of hypertrophic cardiomyopathy associated with CFCS. Such iPSC-hECTs could provide a powerful system for studying species-specific and even patient-specific mechanisms of myocardial dysfunction and for screening new therapeutic strategies for improving the quality of life for patients suffering from HCM and related forms of heart disease.

## Materials and Methods

The human cell lines from which the iPSC lines used in this study were derived from were provided without Protected Health Information. As such, the lines were not identifiable at our institution and, thus, the research was deemed to be not human subjects research subject to internal review board approval from the Program for the Protection of Human Subjects (PPHS) at Mount Sinai. The investigators providing those lines served as “honest brokers.” The investigators providing those lines served as “honest brokers.” The lines were derived from tissue biopsies that were obtained with IRB approval and informed consent from the tissue donors for derivation of and research using these cell lines.

### Generation of hiPSC lines

The iPSC lines were generated as recently explained elsewhere [8]. Human iPSCs were created from patients with the T599R activating *BRAF* mutation [21], a rare [22], but severe [23] form of CFCS, via retroviral-based methods previously reported but with some modification [8]. Briefly, 8x10^5^ dermal fibroblasts were exposed to the retroviruses for 48 hours. After a 24-hour recovery period, the fibroblasts were trypsinized and re-plated on a 10-cm tissue culture treated dish coated with mitotically inactivated mouse embryonic fibroblasts (MEFs). The media was changed every two days using hiPSC maintenance media, consisting of DMEM/F12 (CellGro, Mediatech) supplemented with 20% (v/v) knockout serum replacement (Life Technologies), 5% (v/v) MEF-conditioned medium, penicillin/streptomycin, L-glutamine, non-essential amino acids (Life Technologies), β-mercaptoethanol (Sigma-Aldrich) and 10 ng/ml bFGF (R&D Systems).

After 30 days, hESC-like colonies were mechanically collected and re-plated on 48-well plates coated with MEFs. When needed, colonies were passaged with 0.25% trypsin-EDTA (Life Technologies) and maintained in hiPSC media on the feeder layer. For the first 24 hours after passaging, 10 μM Rho-Kinase “ROCK” inhibitor (Y-27632; Tocris) was also added to the media to enhance survival. Both the wild-type and mutant BRAF T599R iPSC lines will be provided upon request to the authors.

### Differentiation of human cardiac myocytes from iPSC lines

A directed differentiation approach was used to differentiate the mutant and wild-type hiPSCs between passage 20 and 40 to the cardiac lineage using methods reported previously [8] with some modification[Additional reference to paper not yet in press will be added during revision]. In brief, embryoid bodies (EBs) were generated from the hiPSC colonies transferred to a 6-well ultra-low attachment plate (Corning) and maintained at 37°C in 5% CO_2_, 5% O_2_ and 90% N_2_, in differentiation media composed of StemPro^®^34 (Life Technologies) supplemented with 2 mM L-glutamine (Life Technologies), 400 μM monothioglycerol (MTG, Sigma), 50 μg/ml ascorbic acid (Sigma) and 150 μg/ml transferrin (Roche).

Twenty-four hours after EB formation, mesoderm differentiation was achieved by supplementing the differentiation media with 10 ng/ml BMP4 (R&D Systems). The following day, the differentiation media was replaced with differentiation media containing both 10 ng/ml BMP4 (R&D Systems) and 15 ng/ml Activin A (Peprotech) for 72 hours. On day 4, the cardiac mesoderm lineage was promoted by exchanging the differentiation media for differentiation media containing 1.5 μM IWR-1 (Sigma), a potent Wnt inhibitor. After day 8, differentiation media was changed every 5 days with no supplements. Characterization and confirmation of over activation of the RAS/MAPK pathway in the differentiated cells was confirmed via gene expression analysis, and described elsewhere [Josowitz R *et al*., In Revision].

### Live cell sorting

After 30 days of differentiation, the EBs were dissociated overnight with 1 mg/ml collagenase B (Roche) at 37°C. The following morning, the supernatant was removed and 2 ml of TrypLE^™^ (Life Technologies) was added to the EBs for 10 minutes, mixing every 2 minutes. After 10–15 minutes, the reaction was combined with the supernatant containing the collagenase. The cells were then pelleted at 300 x g for 5 min and resuspended in 1 ml of staining buffer (PBS (Sigma-Aldrich) with 10% Fetal Bovine Serum (Atlanta Biologics) and 10 uM ROCK inhibitor (Tocris). Anti-SIRPα-PE-Cy7 (BioLegend) was added at 1:500 dilution to the 1 ml of staining buffer with CD90-FITC (BD Biosciences) at 1:250 dilution and incubated in the dark at 4°C for 1 hour. The cells were then pelleted at 300 x g for 5 min and rinsed 3 times with staining buffer. After the final rinse, the cells were resuspended at 10^6^ cells/ml in staining buffer with 1 μg/ml DAPI (Invitrogen). Live cells were selected by gating to the DAPI^-^ population. Recently, double-sorting using SIRPα and CD90 has revealed the SIRPα^+^/CD90^-^ population to be nearly pure cardiomyocytes, while the SIRPα^-^/CD90^+^ population exhibits a fibroblast-like phenotype [Reference to paper not yet in press will be added during revision]. The PE-Cy7^+^ and FITC^+^ populations were collected separately and recombined at a 3:1 ratio of SIRPα^+^ to CD90^+^ cells in NBS media and reaggregated for 48 hours at 37°C at a concentration of 2 million cells per 10 cm non-tissue culture treated dish to form small aggregates. Gates were determined through the use of control cells stained with the appropriate isotype control antibody independently for each isolation in order to account for variability in both staining and differentiation efficiencies. Cells were sorted on an Aria II cell sorter (BD Biosciences) at 20 PSI.

### Generation of human engineered cardiac tissues (hECTs)

After 48 hours of incubation of the 3:1 cell mixture, the media surrounding the cells was collected and put aside. The tissue culture dish was rinsed with 5 ml of PBS and the rinse transferred to the same tube containing the cell media. Five milliliters of 0.05% trypsin/0.04% EDTA (Cell Detach Kit, PromoCell) were added to the dish and incubated at 37°C for 10 min. The plate was then gently scraped and 5 ml of trypsin neutralization solution (Cell Detach Kit, PromoCell) added to the plate to quench the trypsin. The cell suspension was then combined with the cell media collected earlier. The plate was rinsed once with PBS and transferred to the cell suspension to ensure complete collection of the cells. The entire cell suspension was pelleted at 300 x g for 5 min. The supernatant was removed, the pellet resuspended in 1 ml of NBS media, and transferred to a sterile 1.5 ml centrifuge tube. The cell suspension was centrifuged at 300 x g for 5 minutes and moved to a sterile hood. The supernatant was gently removed and the cell pellet put aside.

The cells were added to the tissue mix as previously described and with some modification [9,24]. Briefly, ice cold bovine Type-I collagen (Sigma-Aldrich) was diluted to 2 mg/ml with 1M NaOH, sterile water, 10x PBS (Sigma) 10x MEM (Sigma) and 2N HEPES pH 9 (Sigma). Ice-cold Matrigel (Corning) was then added to the collagen mix at a concentration of 0.9 mg/ml. The tissue mix was added to the cell pellet to create a final cell concentration of 10 million cells (both SIRPα^+^/CD90^-^ and CD90^+^) per milliliter and a ratio of 1:8:1 (v/v/v) of cells:collagen:matrigel as previously described [9,24].

The cell tissue mixture was pipetted into a custom polydimethylsiloxane (PDMS) mold with 100 μl (10^6^ cells/well) of tissue mix and incubated at 37°C in 5% CO_2_ for two hours to promote tissue compaction and gelling as described previously [9,24]. The tissues were then submerged in NBS media, with half of the media changed very day. Forty-eight hours after tissue construction the PDMS inserts were removed from the mold and tissue function analyzed at day 6 and day 11 ± 1 day.

### H&E staining

To investigate the overall structure of the hECTs, the tissues were fixed in 4% paraformaldehyde, transferred to xylene, rinsed with ethanol, embedded in paraffin and cut in 8-μm-thick sections, which were then stained with hematoxylin and eosin (H&E).

### Functional measurements

Tissue function was assessed as previously described [9,24]. Briefly, tissue contractile function was measured using a high-speed camera (100 frames/s) and a dissecting microscope (Olympus) with custom LabView software (National Instruments) for real-time tracking of deflection of the integrated posts during tissue contraction. Force of contraction was calculated using the measured deflection and the calculated spring constant of the post based on elastic beam-bending theory. Testing was performed inside a biological clean bench to maintain sterility, and with heated NBS media at 37°C. Spontaneous beating frequency and developed twitch force (maximum minus minimum force per twitch) were obtained in the absence of field stimulation, while the force-frequency responses were obtained in the presence of field stimulation at 10 second intervals with 10 seconds of rest between each frequency (0.5 Hz steps). Apart from developed force (DF), the following measurements characterizing the dynamics of the twitch were also measured: twitch duration at 50% of maximal contraction (t_50_) corrected for rate by multiplying by beating frequency to give a time-corrected t_50_ (p_50_); time from 50% to maximal contraction (c_50_); time from maximal contraction to 50% relaxation (r_50_); as well as the maximum rates of contraction (+dF/dt) and relaxation (-dF/dt). Excitation threshold was obtained by pacing at 2 Hz and increasing the stimulation voltage from 1 volt to 12 volts in 0.1 V increments, using carbon electrodes held 20 mm apart, to identify the minimum voltage gradient required to achieve 1:1 capture.

### Gene expression analysis

To determine gene expression of the tissues, total RNA was extracted from the hECTs using Trizol (Life Technologies) and the RNeasy plus isolation kit (Qiagen). RNA was reverse transcribed using oligo-dT primers with the Superscript II Synthesis Kit (Life Technologies). qPCR was completed with Fast SYBR Green Mastermix (Applied Biosystems) according to the manufacturer’s instructions. The primers used were cardiac troponin (*cTnT*), atrial natriuretic peptide (*ANP*), brain natriuretic peptide (*BNP*), and sarcoplasmic/endoplasmic reticulum calcium ATPase 2 (*SERCA2a*). Gene expression was normalized against the housekeeping gene *GAPDH* using the ΔΔ*C*_*t*_ method. The expression level of cardiac genes is reported relative to expression of cardiac-specific cTnT. A StepOne Plus Real-Time PCR System (Applied Biosystems) was used to perform the qPCR and analyzed with StepOne Software v2.2.2.

### Pharmacologic studies

To assess the response of the hiPSC-hECTs to pharmacologic agents, culture media was replaced with DMEM (Sigma-Aldrich) buffered with 20 mM HEPES pH 7.4 (Sigma-Aldrich). Spontaneous beating rate was recorded as described for functional studies, but the tissue was exposed to increasing concentrations of the β-agonist isoproterenol (Hospira) ranging from 1 nM to 5 μM. Each dose was prepared in the buffered DMEM immediately before use, then pipetted directly into the media surrounding the tissue. Data was acquired for 7 minutes per dose to permit sufficient equilibration of the drug in the media. The chronotropic response of each tissue to the drug was measured and normalized so that the minimum spontaneous rate was zero and the maximum was one. The dose response curves were plotted on a semi-log plot and fit with a sigmoid function using Prism 6 software (GraphPad) to identify the EC_50_ of isoproterenol for each tissue type.

### Tissue spontaneous rate variability

The spontaneous rate of each tissue was calculated during 30 seconds of unpaced acquisition. Custom MATLAB (MathWorks) scripts were used to identify each twitch peak and measure the time between peaks. The time-to-next peak and time-to-previous peak for each contraction was measured and plotted against one another to form a cloud of points that represent the variability of the spontaneous rate, similar to methods reported previously [25]. To adjust for differences in average spontaneous rate, the centroid of each cluster for each tissue was calculated using *k*-means clustering where *k* = 1. The centroid coordinates were then projected onto the line of identity and each point divided by the coordinates of the projected point to shift and scale the entire cluster about the (1,1) coordinate. Each tissue was assigned a unique color and the transformed points all plotted with each other. Finally, the relative size of the cloud of transformed points was compared between BRAF-mutant and wild-type hECTs by comparing the average distance of the points from the centroid of the cloud.

### Principal component analysis and cluster analysis

Principal component analysis (PCA) [26] was performed using a custom MATLAB script. The values of the following eight variables, that are relatively independent measures of hECT structure and function, were used as the sort criteria for each tissue: diastolic force (DiF), developed force (DF), cross-sectional area (CA), excitation threshold (ET), maximum capture rate (MCR) and the twitch dynamics parameters c50, r50, and p50. Prior to principal component analysis, the data set was clustered using *k*-means clustering in MATLAB (MathWorks) for *k* = 1 to 12 clusters. The sum of squared distance (SSD) between each point of a cluster with its centroid was calculated for each *k*. The elbow criterion was employed to identify the optimal cluster number by plotting SSD against *k*. The dataset was then clustered to the optimal cluster number. After principal component analysis, the data set was plotted against principal component 1 (PC1) and principal component 2 (PC2) for visualization purposes. The identities of the tissue types were then superimposed upon the plotted points. Thus, tissue identity did not influence the PCA grouping. The loading of each variable onto each principal component reflects its relative contribution to the component [27]. The squared loading values were segregated into an upper regime that constituted “high” loading variables, and a lower regime composed of “low” loading variables.

### Measuring the passive elastic modulus

To determine the passive stiffness of the hECTs, the tissue was transferred to a physiological muscle bath (Scientific Instruments) and connected between a force transducer and stepper motor as previously described [9]. The tissue was perfused with Tyrode’s solution (2.2 mM MgCl_2_, 2.6 mM KCl, 136.8 mM NaCl, 0.4 mM NaH_2_PO_4_, 5.5 mM glucose, 11.9 mM NaHCO_3_ and 3.3 CaCl_2_; Sigma-Aldrich) at 37°C. The force transducer was zeroed after the tissue was attached but with no tension. The stepper motor was then advanced in 0.05-mm increments, and raw force values measured for 15 seconds at each step. Passive force was measured during tissue relaxation (diastolic force) during the last 10 seconds of each step to allow tissue elastic equilibration at each length. Force and motor distance were converted to stress and strain using the cross-sectional area and length of the unloaded tissue, respectively, and the passive elastic modulus was calculated from the slope of a linear model fit between the 5% and 10% strain range.

### Statistics

Descriptive statistics are reported as mean ± standard deviation unless otherwise noted. Comparisons between groups were performed with an unpaired two-tailed Student’s *t*-test for equal variance. Comparisons between day 6 and day 11 were made within each tissue type (BRAF or wild-type) using a paired, two-tailed Student’s *t*-test for equal variance. Analysis of covariance (ANCOVA) was used to test the effect of tissue type on the response of force to pacing frequency in MATLAB (MathWorks). An extra-sum-of-squares F-test was used to compare the dose response curves between each tissue type in Prism 6 (GraphPad). Statistical significance was accepted as p < 0.05. Throughout the study * denotes p < 0.05, ** denotes p < 0.01, *** denotes p < 0.001 between tissue types while † indicates p < 0.05 between day 6 and day 11.

## Results

### BRAF-mutant hiPSC-CMs form hypertrophic cardiac tissues

**Fig 1:**
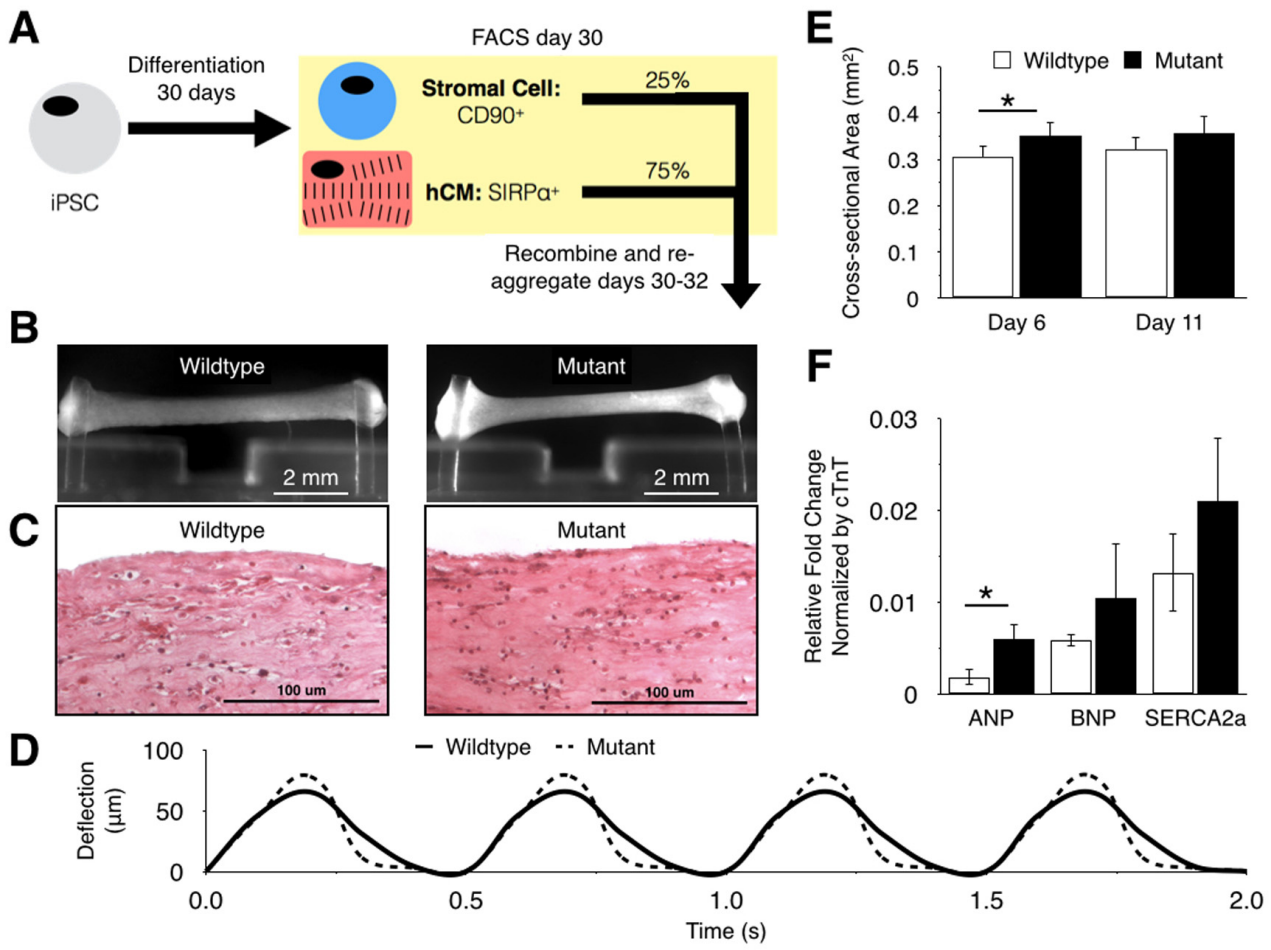
Characterization of wild-type and BRAF iPSC-hECTs. (**A**) Sorting paradigm for construction of defined iPSC-hECTs; (**B**) Photograph of wild-type (left) and mutant (right) tissues after 6 days in culture; (**C**) longitudinal section of wild-type (left) and mutant (right) tissues stained with hematoxylin and eosin after 12 days in culture; (**D**) Post deflections of both tissue types on day 6 at 2 Hz pacing frequency; (**E**) Cross-sectional area (mean±SD) of wild-type (open bars, n = 7) and mutant (filled bars, n = 4) tissues; (**F**) Molecular analysis of mutant and wild-type tissues performed on day 12 (n = 3 for each tissue type).

To address the inconsistent efficiency of differentiating hiPSCs to cardiomyocytes the entire differentiation population was sorted based on SIRPα, a surface marker of cardiomyocytes [28], and THY-1/CD90, a cardiac stromal cell marker [29], and recombined in a 3:1 ratio of SIRPα^+^:CD90^+^ cells that were incubated for 48 hours before being added to the collagen-matrigel solution (Fig 1A) and added to custom PDMS molds to form engineered tissues. During the first few days in culture, both the wild-type and BRAF-mutant hECTs compacted around the PDMS posts to form a thin trabecular muscle-like shape with integrated force sensors at either end (Fig 1B and 1C). By day 6 of culture, the hECTs exhibited spontaneous beating and could be electrically paced, generating measureable post deflections (Fig 1D). The BRAF-mutant hECTs tended to have larger cross-sectional area compared to age-matched WT hECTs (Fig 1E), consistent with the larger size of the BRAF mutant cardiomyocytes in cell culture [Josowitz R *et al*., In Revision]. Gene expression analysis (performed on day 12, after recovery from functional testing described below) showed a significantly increased average expression level of the hypertrophic marker *ANP* and a trend towards increased expression of the hypertrophic marker *BNP* and the calcium ion pump *SERCA2a* in the mutant hECTs (Fig 1F), similar to the cell culture findings [Josowitz R *et al*., In Revision]. Thus, BRAF-mutant hECTs were successfully created, and exhibited several structural and molecular characteristics indicative of a hypertrophic myocardial phenotype.

### BRAF-mutant hECTs exhibit functional characteristics of HCM

**Fig 2:**
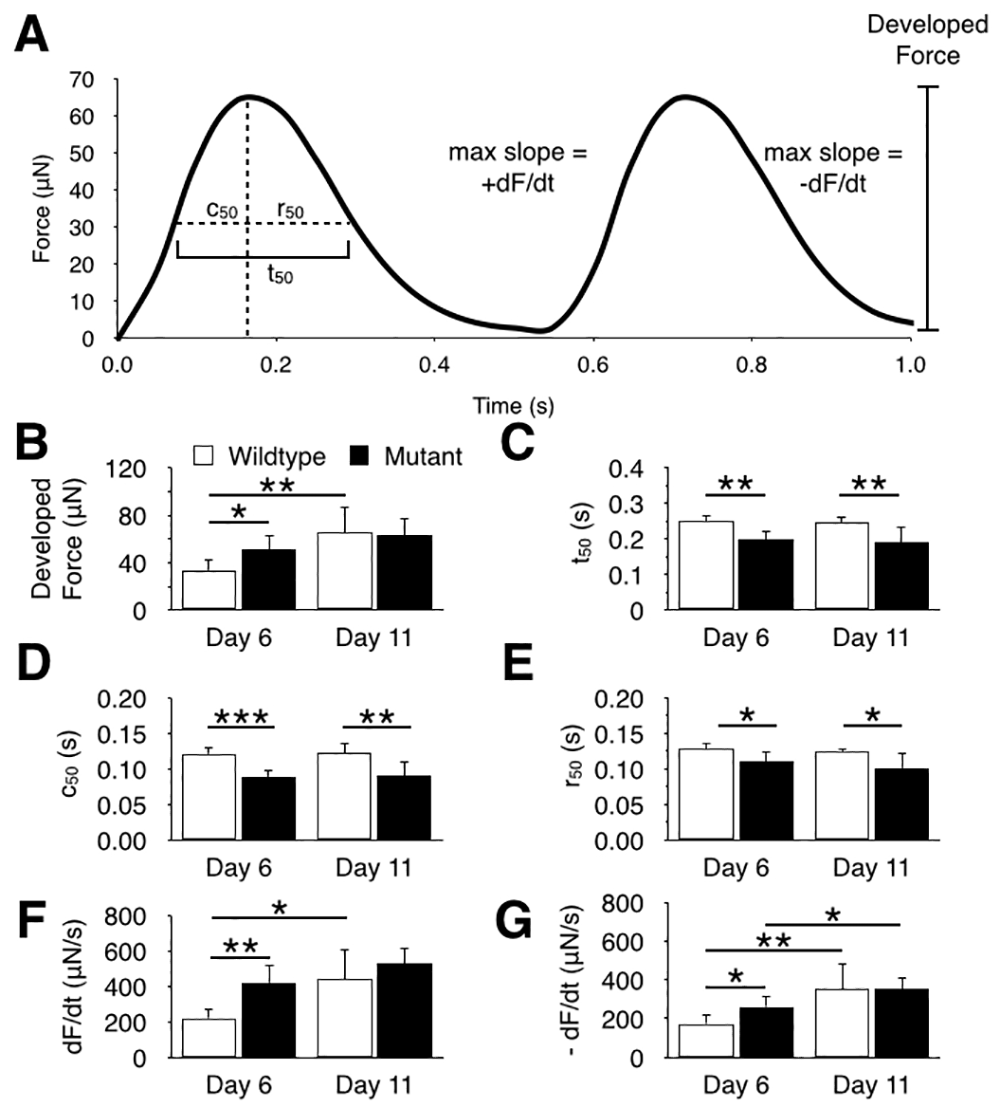
Twitch force characteristics of wild-type and BRAF-mutant hECTs. (**A**) Cartoon of iPSC-derived hECT force data illustrating twitch characteristic parameters. (**B-G**) Mean (±SD) twitch parameters for wild-type (open bars, n = 7) and BRAF mutant (solid bars, n = 5) iPSC-hECTs tested on culture days 6 and 11, including developed force (**B**), 50% twitch duration (**C**), time to 50% contraction (**D**), time to 50% relaxation (**E**) and the maximum rates of contraction (**F**) and relaxation (**G**). * p < 0.05, ** p < 0.01, *** p < 0.001.

To examine the functional differences in contractile performance between BRAF-mutant and WT hECTs, several twitch force characteristics were quantified (Fig 2A). On day 6 of culture, the BRAF-mutant hECTs exhibited significantly greater developed force (Fig 2B) compared to age-matched WT hECTs, indicative of a hypertrophic myocardial phenotype. The mutant tissues also exhibited a shorter twitch duration (Fig 2C), reflecting both shorter contraction (Fig 2D) and relaxation (Fig 2E) phases of the twitch. The maximum rates of contraction (Fig 2F) and relaxation (Fig 2G) were also significantly higher for BRAF-mutant tissues at day 6. With the notable exception of developed force and the rates of contraction/relaxation, these differences in twitch characteristics were largely maintained in experiments repeated on day 11, reflecting accelerated twitch dynamics in BRAF-mutant hECTs that could portend a tendency toward arrhythmias, as observed clinically in HCM patients. Thus, BRAF-mutant hECTs emulated functional characteristics of HCM, although some distinctions from WT hECTs varied with time in culture.

### BRAF-mutant iPSC-derived hECTs display altered electrical properties

**Fig 3:**
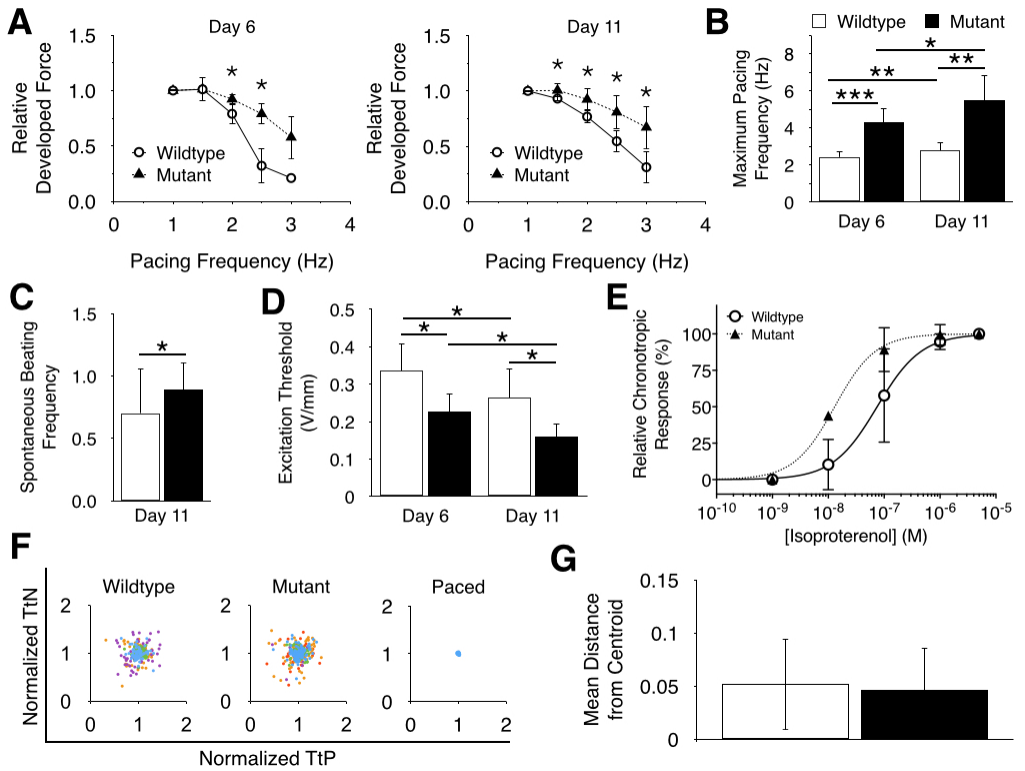
Electrical properties of of wild-type and BRAF-mutant hECTs. (**A**) Developed force versus frequency relationship of iPSC-derived hECTs at culture day 6 (left) and day 11 (right); (**B**) maximum pacing frequency of wild-type and mutant tissues; (**C**) spontaneous beating frequency; (**D**) The minimum voltage necessary for pacing for both tissues; (**E**) Relative chronotropic response to isoproterenol (normalized to baseline beating frequency) with fitted nonlinear regression model. (**F**) Twitch rate variability plots of each tissue type with a paced control for comparison. Each color represents a different tissue. (**G**) Quantification of twitch rate variability by the mean distance of each point on the twitch rate variability plots from each cluster centroid. Error bars represent standard error (**A**) or standard deviation (**B-G**) Open symbols and bars are wild-type hECTS (n = 7), filled symbols and bars are BRAF-mutant hECTs (n = 5). * p < 0.05, ** p < 0.01, *** p < 0.001.

Since the dynamics of the twitch are mechanical consequences of the electrical signals within the tissue, we hypothesized that the BRAF-mutant hECTs would exhibit altered electrical properties compared to the WT hECTs. Both tissue types exhibited a negative force frequency relationship (Fig 3A), but mutant tissues were less sensitive to pacing frequency. The interaction term for an analysis of covariance was significant at day 6 (p < 0.01) and day 11 (p < 0.05), confirming that the force-frequency relationship was affected by the tissue type. Additionally, the mutant tissues could be paced at a higher maximum frequency than WT hECTs at day 6 and day 11 (Fig 3B), and they exhibited significantly higher spontaneous beating frequencies (Fig 3C), consistent with the faster twitch dynamics described above. BRAF-mutant hECTs also possessed a significantly lower excitation threshold, requiring a lower voltage for pacing than their wild-type counterparts on both days of testing (Fig 3D). Increased excitability of BRAF-mutant hECTs was also evident in the chronotropic response to pharmacologic stimulation of beta-adrenergic effects, whereby the spontaneous beating frequency showed a positive dose response to isoproterenol treatment, with a significantly lower EC_50_ of 13 nM (95% confidence interval: 10–16 nM) compared to 75 nM in WT-hECTs (95% confidence interval: 40–140 nM) (p <0.0001, Fig 3E). Despite these indications of increased arrhythmogenic potential in BRAF-mutant hECTs, intrinsic variability in the spontaneous beating rate (Fig 3F) did not appear greater in BRAF-mutant hECTs than in WT tissues (Fig 3G). Further electrophysiological testing using a programmed electrical stimulation protocol [30] may be required to assess the true arrhythmogenic potential of hECTs.

### Investigating longitudinal changes in hECT functional phenotype

**Fig 4:**
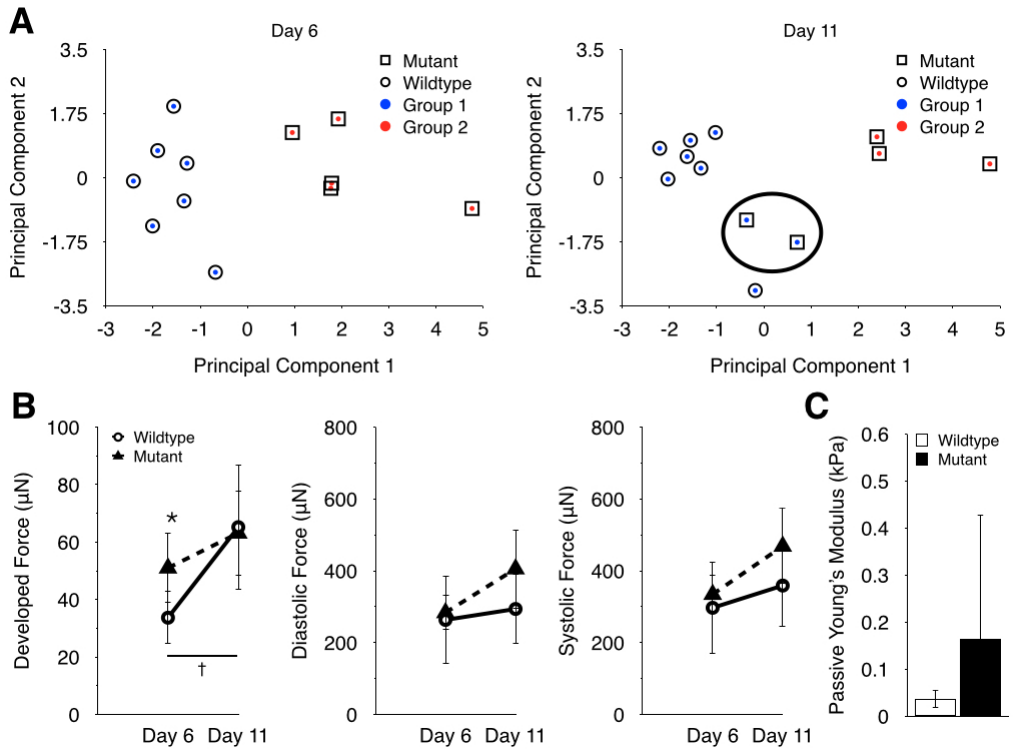
Investigating longitudinal changes in hECT functional phenotype. (**A**) Principal component analysis and k-means clustering of tissues paced at 2 Hz on day 6 (left) and day 11 (right). (**B**) Developed force of the wild-type tissues increased significantly over day 6 and day 11, while mutant tissues did not. The lack of a significant change from day 6 to day 11 appeared to be due to a greater increase in diastolic force of mutants from day 6 to 11 than wild-type tissues, while systolic force increased approximately by the same amount for both tissue types. (**C**) Passive Young’s modulus determined by uniaxial stretch measurements on days 12–15 (n = 4 per tissue type). * p < 0.05 between tissues types, † p < 0.05 between day 6 and day 11 for wild-type tissues.

To gain insight into the key factors that distinguished BRAF-mutant hECTs from WT hECTs, principal component analysis was combined with *k*-means cluster analysis, incorporating eight physical and functional parameters measured on both days of hECT testing. Plotting SSD versus *k* and applying the elbow method of determining optimal cluster number, the data were clearly grouped into two clusters (S1 Fig), consistent with having two distinct hECT tissue types. After the clusters were determined, the identity of each tissue type was revealed, providing an unbiased approach to examine grouping within the data. On testing day 6, the two groups correctly self-segregated into mutant and wild-type tissues (Fig 4A, left). However, on testing day 11, two of the BRAF-mutant hECTs were more closely identified with the wild-type cluster (Fig 4A, right, circle). To understand the factors driving this change, we examined the contribution of each of the eight parameters to each principal component.

The principal components (PCs) are ranked such that PC1 is the largest contributor to the variation in the data, and PC8 is the smallest contributor (S1 Fig, S1 and S2 Tables). Therefore, in a mixed population of mutant and wild-type tissues, it is likely that the first few principal components will be primarily associated with differences between BRAF-mutant and WT hECTs. As shown in Table 1, more than 80% of the total variance on day 6 was explained by the first two principal components, where PC1 was dominated by parameters related to twitch dynamics (ET, MCR, c50, r50 and p50), and PC2 was dominated by contractile function parameters (DF and DiF). On Day 11 (Table 2), the functional distinction between PC1 and PC2 was less clearly defined, and notably the diastolic force (DiF) rose to a more prominent role as a component of PC1, motivating a more detailed examination of the twitch force data.

**Table 1:** Summary of component loadings on the principal components that explain 95% of the variance on experimental day 6.

Top variable loadings on principle components	Orthogonal Coefficient Value	Variance Explained	
		Incremental	Cumulative
**Component 1: Twitch Dynamics**		60.7%	60.7%
Cross-sectional Area	0.347		
(Excitation Threshold)	-0.362		
Maximum Capture Rate	0.442		
(c50)^a^	-0.418		
(r50)	-0.395		
(p50)	-0.417		
**Component 2: Contractile Function**		20.7%	81.4%
Developed Force	0.606		
Diastolic Force	0.606		
**Component 3**		10.3%	91.71%
(Diastolic Force)	-0.612		
(Excitation Threshold)	-0.514		
**Component 4**		4.39%	96.1%
(Developed Force)	-0.598		
(Excitation Threshold)	-0.427		

The first principal component is composed mostly of twitch dynamics parameters while the contraction force of the tissue is present in component 2.

^a^Parentheses denote negative loading on the component.

**Table 2:** Summary of component loadings on the principal components that explain 95% of the variance on experimental day 11.

Top variable loadings on principle components	Orthogonal Coefficient Value	Variance Explained	
		Incremental	Cumulative
**Component 1: Twitch Dynamics**		59.2%	59.2
^†^Diastolic Force	0.332		
Maximum Capture Rate	0.452		
(c50)	-0.416		
(r50)	-0.430		
(p50)	-0.428		
**Component 2: Contractile Function**		21.9%	81.2
(Developed Force)	-0.629		
^†^(Cross-sectional Area)	-0.554		
**Component 3**		10.0%	91.2
(Diastolic Force)	-0.559		
^†^(Developed Force)	-0.527		
^†^Cross-sectional Area	0.438		
(Excitation Threshold)	-0.431		
**Component 4**		5.55%	96.7
Diastolic Force	0.541		
(Excitation Threshold)	-0.406		
^†^c50	0.430		

The first principal component is composed mostly of twitch dynamic parameters and, unlike day 6, diastolic force.

^†^Indicates a difference between experimental day 6 and day 11.

Developed force (DF) is the difference between systolic force (SyF) and diastolic force (DiF) for each twitch. While the developed force of the WT hECTs significantly increased from day 6 to day 11, the increase in DF for the BRAF-mutant hECTs was not statistically significant (Fig 4B). Whereas both tissue types showed similar increasing trends in systolic force from day 6 to 11, the BRAF-mutant hECTs also showed an increase in diastolic force exceeding that in WT hECTs, which accounts for the attenuated increase in developed force in the mutant tissues. This may also explain why the principal component analysis found DiF to be a more prominent factor explaining the variability between tissue groups at day 11 than at day 6.

The elevated diastolic force in BRAF-mutant hECTs could reflect impaired relaxation of the cardiomyocytes or elevated passive stiffness of the tissue. Impaired relaxation causes diastolic force to increase during high frequency pacing. While DiF increased with frequency for both tissue types, the BRAF-mutant hECT response was significantly less sensitive to frequency than the WT-hECTs on both days of testing (S2 Fig), as confirmed by the type*frequency interaction effect in ANCOVA (p < 0.001). This is consistent with the lower r_50_ and larger -dF/dt twitch characteristics indicating faster relaxation in BRAF-mutant vs. WT hECTs. Thus, impaired relaxation is unlikely to be responsible for the elevated DiF in BRAF-mutant hECTs.

When WT and BRAF-mutant hECTs were subjected to uniaxial stretch testing on a physiologic muscle bath apparatus, the slope of the resulting diastolic stress-strain curve yielded an apparent Young’s modulus for the passive tissue that was higher on average in BRAF-mutant hECTs (0.164±0.264 kPa, n = 4) compared to WT tissues (0.036±0.019 kPa, n = 4) (Fig 4C). Note that the large standard deviation in the BRAF-mutant hECTs is due to one particularly stiff tissue. Consequently, although the power of the data was not sufficient to achieve statistical significance, the trend suggests the observed changes in diastolic force in the BRAF-mutant hECTs may partly reflect an elevated passive stiffness of the mutant cardiac tissues, which is a common characteristic of HCM.

## Discussion

A number of previous studies have described the construction of engineered cardiac tissues from human embryonic stem cell-derived cardiomyocytes [9,25,31–33], while only a few papers have described hECTs created from human iPSCs [31,32,34,35]. One explanation is that iPSC lines are notoriously variable [36] and, generally, differentiate at lower efficiencies than embryonic stem cells.

We have overcome this limitation of hiPSCs through the use of live cell sorting and recombining defined populations of cardiomyocytes and stromal cells to accommodate differences in both differentiation efficiency and also variability in cardiac differentiation capacity between iPSC lines. Other groups have utilized genetic reporter lines to create similarly defined tissues, but we are among the first to combine two surface markers to collect the cardiomyocytes and stromal cells prior to construction of hECTs. In this study, three iPSC lines were used—two lines derived from separate healthy patients and one line derived from a patient with CFCS. Notably, a second BRAF mutant line was also investigated as a means of creating the mutant tissues, but this second line failed to form functional hECTs. The inability for some iPSC lines to form functional tissues is puzzling and warrants careful consideration when proceeding with hiPSC-hECT studies.

Other examples of modeling disease in ECTs have been reported, but, to our knowledge, this is the first example of a human model of inherited HCM. Ma *et al*. reported the formation of hECTs from patients with long-QT syndrome type 3 [34]. While the study was among the first to create mutant hECTs, the disease primarily causes abnormalities in electrophysiology; so, unlike HCM, a model system that allowed measurement of contractile force was not required. An engineered model of an inherited cardiomyopathy from patients with Barth syndrome, a mitochondrial disorder, has also been reported [37], but the heart-on-chip technology used in the report is essentially two-dimensional and lacks the direct twitch force measurement capability and enhanced biofidelity of the third dimension present in our system. A model of pathologic hypertrophy has been reported in rat ECTs [10] but the hypertrophy in that instance was induced via mechanical loading and not inherited. Thus, our study is unique in that we report the first example of modeling human HCM in 3-D hECTs using a genetic form of the disease. Future studies examining other mediators of the hypertrophic response, including micro-RNAs, could be analyzed to further categorize HCM in the BRAF hECTs.

Our results demonstrate that abnormal BRAF activation leads to significant alterations in cardiac tissue function in hECTs. In total, the results suggest that BRAF hyper-activation in hECTs leads to a hypertrophic phenotype, including accelerated twitch dynamics with overall shorter twitches and differences in the response to pacing frequency between wild-type and mutant hECTs. The enhanced twitch force and larger cross-sectional area observed at the early time point may reflect the larger size and increased sarcomeric organization observed in the single cells [Josowitz R *et al*., In Revision]. The BRAF-mutant tissues also exhibited increased sensitivity to isoproterenol. The resultant shorter twitches, and alterations in response to both electrical and beta-adrenergic stimuli suggests a potentially arrhythmogenic substrate due to BRAF hyper-activation.

Previous studies have attempted to create models of hypertrophy in ECTs. Using rat ECTs, transiently elevated afterload during culture induced evidence of pathologic hypertrophy, including enhanced expression of the hypertrophic markers *ANP* and *BNP* [38]. Interestingly, in the rat study, the loaded tissues displayed decreased rates of contraction and relaxation while in our study, these rates were elevated in the mutant tissues. Thus, the altered twitch dynamics may reflect a species-dependent phenomenon or an inherent effect of the *BRAF* mutation rather than a secondary effect of the hypertrophic response. The differences between the rat study and the study presented here may also represent differences in the stage of HCM progression as the rat study may be more representative of an early form of the disease while the study presented herein, given its inherited nature, may represent a later, chronic form of the disease. Thus, the increased afterload and the inherited BRAF models are not mutually exclusive and may, in fact, represent different stages of HCM progression. An inherited cardiomyopathy has been modeled with heart-on-chip technologies using iPSCs from patients with Barth syndrome, a mitochondrial disorder, where irregular sarcomeric organization and corresponding reduced twitch stress was observed [37], differing from our model where greater stress was observed. However, in cardiomyocytes derived from patients with Noonan syndrome with multiple lentigines, another RASopathy, larger cell size and greater sarcomeric organization was observed [8], consistent with our findings of enhanced tissue function.

By utilizing principal component analysis combined with cluster analysis and comparing two time points of tissue culture, we identified aspects of tissue function that contributed to variability, both genotypically and longitudinally. The altered twitch dynamics between control and mutant tissues played a dominant role in distinguishing tissue types at both time points, which may reflect a functional consequence of the dysregulated calcium handling observed in HCM [2,39] and Ras activation [40,41] and also observed in the purified BRAF-mutant cells [Josowitz R *et al*., In Revision]. Other studies have found that conduction velocity increases with shorter twitch duration[42], suggesting that the shorter T_50_ observed in the BRAF mutant hECTs may represent enhanced conduction throughout the tissue. Mechanistically, the activity of hERG K^+^ channels has been shown to be regulated by BRAF [43], which could potentially alter the dynamics of the twitch in the BRAF-mutant hECTs. One potential limitation of the use of PCA in this study is the low power associated with our PCA. Nevertheless, the PCA was informative since it directed investigation into the contribution of diastolic force to tissue phenotype over time. Analysis revealed that the lost difference in developed force at day 11 was due to the much larger increase in diastolic force from day 6 to 11 for the mutant compared to the wild-type hECTs. These observations also point out that the functional phenotype of engineered tissues can vary with time in culture, which may be particularly noteworthy when developing new disease models.

## Conclusions

In conclusion, we have created the first diseased human ECTs from hiPSC-derived cardiomyocytes that reveal functional characteristics of BRAF mutant-mediated HCM, including altered contractile properties, accelerated twitch dynamics, and increased sensitivity to beta-adrenergic stimulation, which may reflect an enhanced susceptibility to arrhythmias. We also report tissue function at multiple time points to assess longitudinal changes in phenotype that may be important in developing iPSC-hECTs for disease modeling applications. Cluster analysis provided an unbiased method to examine tissue grouping that, when combined with principal component analysis, helped direct identification of key factors that drive the tissue phenotype, which may also be suitable parameters to monitor in screening applications. Thus, such iPSC-hECTs created using cells from healthy and diseased human patients, hold the intriguing potential to create next generation model systems for developing future therapeutics for patients suffering from HCM and related disorders.

## Supporting Information

S1 Fig Elbow plot analysis of tissue physical and functional characteristics. The decrease in sum of squared distance, defined as the sum of the squares of each point from their group centroid, dramatically changes at *k* = 2 clusters forming an “elbow” and supporting two clusters as the optimum at both day 6 (**A**) and day 11 (**B**).
(DOCX)

S2 Fig Wild-type tissue diastolic force changes more with frequency than mutant tissues at both day 6 and day 11 of pacing. (**A**) Representative twitch tracing for wild-type (top) and mutant (bottom) at both day 6 (left) and day 11 (right) of pacing from 1–2.5 Hz; (**B**) Force-frequency relationship of hiPSC-hECT diastolic force relative to the diastolic force at 1 Hz at both day 6 (left) and day 11 (right); (C) Force-frequency relationship of hiPSC-derived hECT systolic force relative to the starting systolic force at 1 Hz at both day 6 (left) and day 11 (right). At both day 6 and day 11, the change in diastolic force versus frequency for the mutant is much less than that of the wild-type. Error bars represent standard error. * p < 0.05, ** p < 0.01 between mutant and wild-type relative forces.
(DOCX)

S1 Table Orthogonal principal component coefficients on day 6 of experimentation. (DOCX)

S2 Table Orthogonal principal component coefficients on day 11 of experimentation. (DOCX)
