# Supplementary material for: Human Engineered Cardiac Tissues Created Using Induced Pluripotent Stem Cells Reveal Functional Characteristics of BRAF-Mediated Hypertrophic Cardiomyopathy
Source: PLoS One. 2016 Jan 19;11(1):e0146697. doi: 10.1371/journal.pone.0146697 (PMC4718533; doi:10.1371/journal.pone.0146697)
Supplement: S1 Fig — The decrease in sum of squared distance, defined as the sum of the squares of each point from their group centroid, dramatically changes at k = 2 clusters forming an “elbow” and supporting two clusters as the optimum at both day 6 (A) and day 11 (B). (DOCX) [file pone.0146697.s001.docx]

**
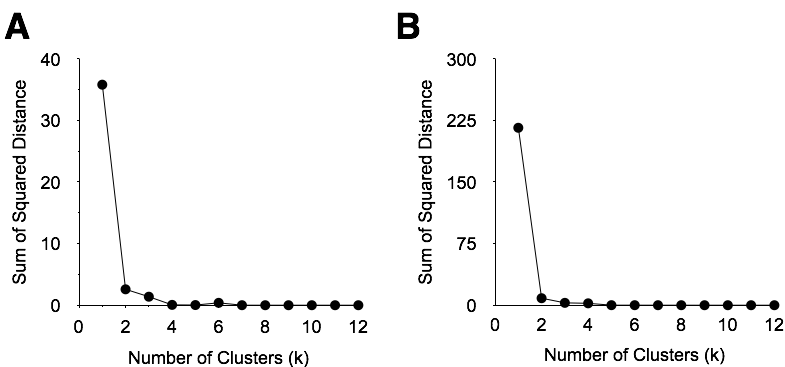
**

**S1 Fig. Elbow plot analysis of tissue physical and functional characteristics.** The decrease in sum of squared distance, defined as the sum of the squares of each point from their group centroid, dramatically changes at *k* = 2 clusters forming an “elbow” and supporting two clusters as the optimum at both day 6 (**A**) and day 11 (**B**).
