# Supplementary material for: Human Engineered Cardiac Tissues Created Using Induced Pluripotent Stem Cells Reveal Functional Characteristics of BRAF-Mediated Hypertrophic Cardiomyopathy
Source: PLoS One. 2016 Jan 19;11(1):e0146697. doi: 10.1371/journal.pone.0146697 (PMC4718533; doi:10.1371/journal.pone.0146697)
Supplement: S2 Fig — (A) Representative twitch tracing for wild-type (top) and mutant (bottom) at both day 6 (left) and day 11 (right) of pacing from 1–2.5 Hz; (B) Force-frequency relationship of hiPSC-hECT diastolic force relative to the diastolic force at 1 Hz at both day 6 (left) and day 11 (right); (C) Force-frequency relationship of hiPSC-derived hECT systolic force relative to the starting systolic force at 1 Hz at both day 6 (left) and day 11 (right). At both day 6 and day 11, the change in diastolic force versus frequency for the mutant is much less than that of the wild-type. Error bars represent standard error. * p < 0.05, ** p < 0.01 between mutant and wild-type relative forces. (DOCX) [file pone.0146697.s002.docx]

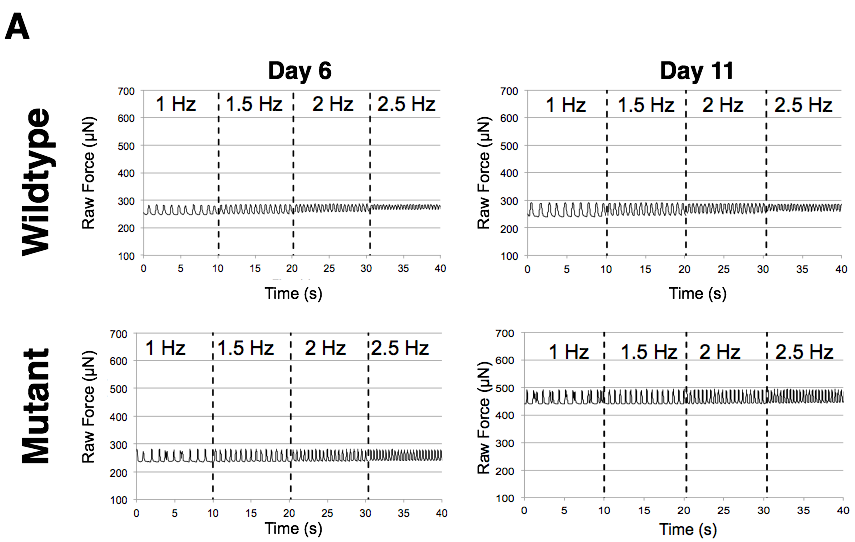


**
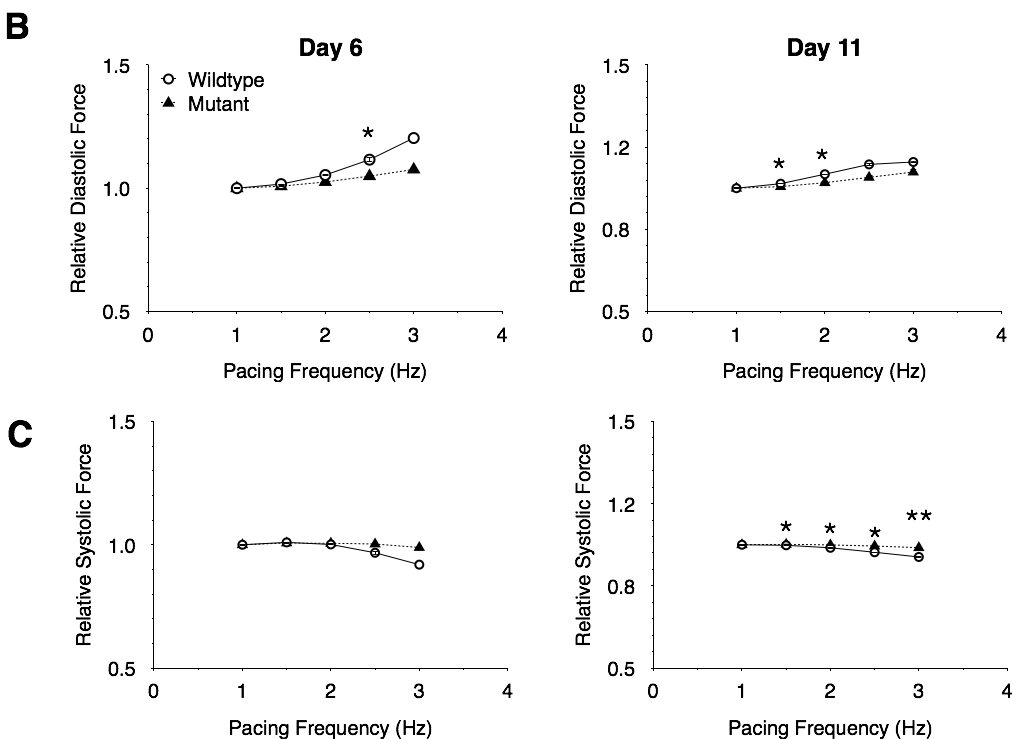
**

**S2 Fig. Wild- type tissue diastolic force changes more with frequency than mutant tissues at both day 6 and day 11 of pacing.** (**A**) Representative twitch tracing for wild type (top) and mutant (bottom) at both day 6 (left) and day 11 (right) of pacing from 1-2.5 Hz; (**B**) Force-frequency relationship of hiPSC-hECT diastolic force relative to the diastolic force at 1 Hz at both day 6 (left) and day 11 (right); (C) Force-frequency relationship of hiPSC-hECT systolic force relative to the starting systolic force at 1 Hz at both day 6 (left) and day 11 (right). At both day 6 and day 11 the change in diastolic force versus frequency for the mutant is much less than that of the wild type. Error bars represent standard error. * p < 0.05, ** p < 0.01 between mutant and wild type relative forces.
