# Supplementary material for: Human Engineered Cardiac Tissues Created Using Induced Pluripotent Stem Cells Reveal Functional Characteristics of BRAF-Mediated Hypertrophic Cardiomyopathy
Source: PLoS One. 2016 Jan 19;11(1):e0146697. doi: 10.1371/journal.pone.0146697 (PMC4718533; doi:10.1371/journal.pone.0146697)
Supplement: S1 Table — (DOCX) [file pone.0146697.s003.docx]

| **Factor** | **Principal Component Number** | | | | | | | |
| --- | --- | --- | --- | --- | --- | --- | --- | --- |
|  | **PC 1** | **PC 2** | **PC 3** | **PC 4** | **PC 5** | **PC 6** | **PC 7** | **PC 8** |
| DiF | -0.035 | **0.606** | **-0.612** | 0.413 | 0.261 | -0.083 | 0.106 | -0.018 |
| DF | 0.218 | **0.606** | 0.182 | **-0.598** | 0.159 | -0.023 | -0.409 | 0.034 |
| CA | **0.347** | 0.360 | 0.232 | 0.199 | **-0.737** | -0.220 | 0.252 | -0.030 |
| ET | **-0.362** | 0.030 | **-0.514** | **-0.427** | **-0.536** | 0.365 | 0.018 | 0.003 |
| MCR | **0.442** | 0.059 | 0.082 | 0.173 | 0.081 | **0.866** | 0.088 | -0.007 |
| c50 | **-0.418** | 0.150 | 0.255 | 0.375 | -0.160 | 0.169 | **-0.547** | -0.494 |
| r50 | **-0.395** | 0.260 | 0.342 | -0.204 | 0.206 | 0.102 | **0.671** | -0.339 |
| p50 | **-0.417** | 0.205 | 0.293 | 0.190 | -0.037 | 0.145 | -0.023 | **0.799** |
| *% Incr. Variance* | *60.714* | *20.707* | *10.282* | *4.389* | *3.209* | *0.481* | *0.205* | *0.012* |
| *% Cum. Variance* | *60.714* | *81.421* | *91.703* | *96.092* | *99.301* | *99.782* | *99.988* | *100* |

**S1 Table. Orthogonal principal component coefficients on day 6 of experimentation.**

DiF = Diastolic Force; DF = Developed Force; CA = Cross-sectional Area; ET = Excitation Threshold; MCR = Maximum Capture Rate; c50 = Contraction time at 50% of maximal twitch; r50 = Relaxation time at 50% of maximal twitch; p50 = t50 multiplied by beating frequency; % Incr. Variance = Incremental percent of total variance accounted for; % Cum. Variance = Cumulative percent of total variance accounted for. Bolded loading values indicate the high loading factors.
