# Supplementary material for: Human Engineered Cardiac Tissues Created Using Induced Pluripotent Stem Cells Reveal Functional Characteristics of BRAF-Mediated Hypertrophic Cardiomyopathy
Source: PLoS One. 2016 Jan 19;11(1):e0146697. doi: 10.1371/journal.pone.0146697 (PMC4718533; doi:10.1371/journal.pone.0146697)
Supplement: S2 Table — (DOCX) [file pone.0146697.s004.docx]

| **Factor** | **Principal Component Number** | | | | | | | |
| --- | --- | --- | --- | --- | --- | --- | --- | --- |
|  | **PC 1** | **PC 2** | **PC 3** | **PC 4** | **PC 5** | **PC 6** | **PC 7** | **PC 8** |
| DiF | **0.332** | -0.203 | **-0.559** | **0.541** | 0.316 | -0.292 | -0.242 | -0.006 |
| DF | -0.044 | **-0.629** | **-0.527** | -0.345 | -0.281 | 0.312 | 0.170 | 0.0175 |
| CA | 0.204 | **-0.554** | **0.438** | -0.345 | **0.543** | -0.176 | -0.116 | -0.0327 |
| ET | -0.319 | 0.360 | **-0.431** | **-0.406** | **0.598** | -0.0854 | 0.23208 | -0.00029 |
| MCR | **0.452** | 0.015 | 0.0862 | 0.176 | 0.0833 | 0.0382 | **0.865** | -0.0178 |
| c50 | **-0.416** | -0.177 | 0.114 | **0.430** | 0.311 | **0.527** | 0.0584 | -0.469 |
| r50 | **-0.430** | -0.218 | 0.0243 | 0.0723 | -0.232 | **-0.706** | 0.257 | -0.379 |
| p50 | **-0.428** | -0.218 | 0.106 | 0.289 | 0.106 | -0.0412 | 0.166 | **0.796** |
| *% Incr. Variance* | *59.219* | *21.931* | *10.047* | *5.5497* | *2.3516* | *0.68732* | *0.21175* | *0.002981* |
| *% Cum. Variance* | *59.219* | *81.15* | *91.197* | *96.746* | *99.098* | *99.785* | *99.997* | *100* |

**S2 Table. Orthogonal principal component coefficients on day 11 of experimentation.**

DiF = Diastolic Force; DF = Developed Force; CA = Cross-sectional Area; ET = Excitation Threshold; MCR = Maximum Capture Rate; c50 = Contraction time at 50% of maximal twitch; r50 = Relaxation time at 50% of maximal twitch; p50 = t50 multiplied by beating frequency; % Incr. Variance = Incremental percent of total variance accounted for; % Cum. Variance = Cumulative percent of total variance accounted for. Bolded loading values indicate the high loading factors.
